# The Utility of Endoscopic Ultrasound Guided Fine Needle Aspiration in the Diagnosis of Infectious Diseases—Report of Three Cases

**DOI:** 10.1155/2013/512182

**Published:** 2013-12-10

**Authors:** Mauro Ajaj Saieg, Felipe Yazawa, Manuela Horta, Lucio G. Rossini, Mabel Tatty Medeiros Fracassi, Fabíola Del Carlo Bernardi

**Affiliations:** ^1^Department of Pathology, Santa Casa Medical School, Rua Doutor Cesário Mota Junior 112, 01221-020 São Paulo, Brazil; ^2^Santa Casa Medical School, Rua Doutor Cesário Mota Junior 112, 01221-020 São Paulo, Brazil; ^3^Department of Endoscopy, Santa Casa Medical School, Rua Doutor Cesário Mota Junior 112, 01221-020 São Paulo, Brazil

## Abstract

Endoscopic ultrasound-guided fine needle aspiration (EUS-FNA) is a fast and minimally invasive methodology with a crucial impact on patients' management. It has an important and established role in the diagnosis and staging of mediastinal and abdominal malignancies, but little is discussed in the literature on the usefulness of this technique in the diagnosis of infectious diseases. In the current report, we present three different cases where EUS was essential for reaching the diagnosis of tuberculosis and paracoccidiodomicosis in cases otherwise seen as malignant. In conclusion, EUS was successful not only in obtaining enough cells for morphological analysis, but also for the production of cell blocks and assessment of the presence of the microorganisms by special stains. EUS allied to fine needle biopsy was an important tool in determining diagnoses of enlarged lymph nodes, revealing the diagnosis of infectious diseases in cases otherwise seen as malignant. The wide use of this methodology in cases such as those reported here cannot only rule out malignancy, but also aid critically ill patients by installing early proper therapy without the need for aggressive interventions.

## 1. Introduction

Endoscopic ultrasound guided fine needle aspiration (EUS-FNA) was introduced to clinical practice 30 years ago and has recently shown to be of important value as an implement to modern clinical practice, with a decisive impact on patients' management, but little is discussed in the literature on the usefulness of this technique for the diagnosis of infectious diseases, with few anecdotal reports [1–6] (Table 1).

In the current report, we present three different clinical scenarios where EUS was crucial to reaching the diagnosis of infectious diseases, obtaining sufficient material not only for morphological analysis but also for subsequent stains in order to detect the presence of microorganisms.

## 2. Cases

*The 1st Case*. A 25-year-old, female, African Brazilian arrived at the emergency service referring abdominal pain of high intensity, starting 15 days ago. The pain was accompanied by fever, vomits, and one episode of diarrhea. The patient was HIV positive, in regular use of antiretroviral (ART) for the past 2 years, and had a previous history of pulmonary tuberculosis.

At physical examination, the patient was mildly pale, with a heart rate of 76 bpm, respiratory rate of 14 ipm, and blood pressure of 110 × 80 mmHg. The abdomen was painful at palpation, especially in the right hipocondrium, but showed no signs of peritonitis (i.e., tenderness to palpation or increased abdominal wall rigidity). Ultrasound and computed tomography (CT) of the abdomen showed hepatosplenomegaly with thrombosis and partial dilation of the distal part of the splenic vein. There was also a notable enlargement of the peripancreatic, periaortic, and intrahaortic lymph nodes.

**Figure 1:**
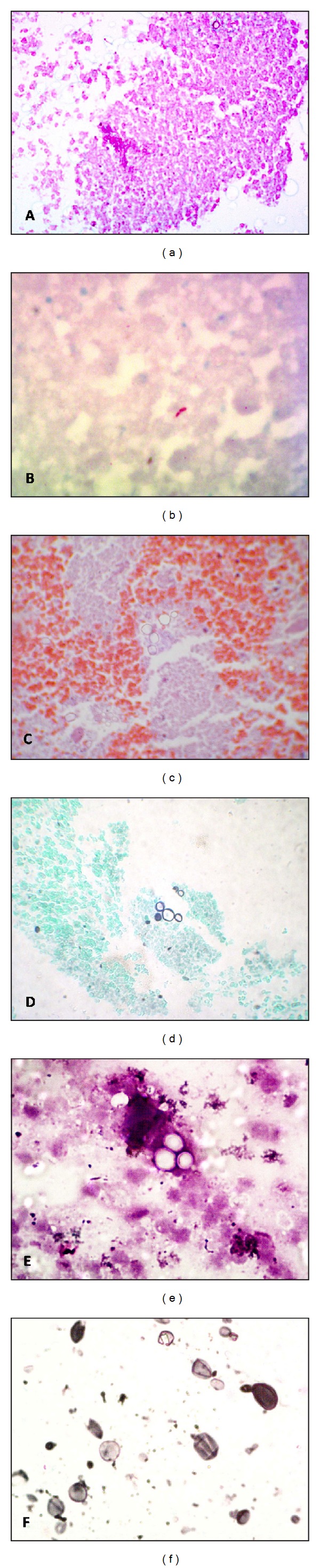
(a) Cell block section showing necrosis (H&E, 40x). (b) Ziehl-Neelsen showing fast-acid bacilli, compatible with tuberculosis. (c) and (e) H&E section from cell blocks from cases 2 and 3 showing fungal structures compatible with *Paracoccidioides brasiliensis*. (d) and (f) Grocott staining of the cases pictured in (c) and (e) with evidence of the blasts with multiple exofitic sporulation.

EUS was performed using a sectorial probe of 7.5 MHz. It showed multiple round, hypoecogenic, homogeneous lymph nodes, the larger ones with a diameter of 1.5 cm, localized in the hepatic and splenic hilum and peripancreatic region. A fine needle aspiration (FNA) was performed in the peripancreatic lymph node using a 22-gauge needle. The aspirate was smeared onto a slide and the remaining material in the syringe was rinsed in formaldehyde and sent to the lab for cell block production (centrifugation at 12,000 rpm for 10 minutes with 20 mL of agar gel, followed by paraffin embedding of the pellet). Cytopathological analysis of the smear was mainly necrotic and inconclusive due to the lack of viable cells in the sample, but the cell block showed small fragments of tissue, with formation of structures suggestive of granulomas, amidst in necrosis. The Ziehl-Neelsen stain showed acid-fast bacilli (Figures 1(a) and 1(b)), and the final diagnosis was nodal mycobacteriosis.

*The 2nd Case*. A 40-year-old male reported weakness, loss of 30 kg, and pain on the left shoulder. The patient was under treatment for diabetes and reported alcohol abuse. The patient had a CT scan showing hypodense, heterogeneous lesions in both adrenal glands, measuring 4,0 × 3,8 cm (right) and 5,0 × 4,0 cm (left). Clinical diagnosis was primary adrenal insufficiency and therapy was initiated with prednisone, with initial good outcome. After six months, however, the patient showed remission of symptoms and was referred to our service with arterial hypotension and worsening of general clinical status.

New clinical hypotheses included secondary adrenal insufficiency due to tuberculosis, lymphoma, or paracoccidioimycosis and a magnetic resonance imaging (MRI) was performed, followed by a EUS with biopsy of the left adrenal gland. Histological examination of the fragments obtained showed areas of necrosis with scant tissue. Special stainings for detection of microorganisms were performed and the Grocott silver impregnation confirmed the presence of fungi in the form of blasts, with morphological characteristics compatible with *Paracoccidioides brasiliensis* (Figures 1(c) and 1(d)). The patient was treated with amphotericin-B with complete remission after 60 days.

*The 3rd Case*. A 57-year-old female patient arrived at the service referring abdominal pain and jaundice three months before and a gradual weight loss starting 6 months ago (total of 12 kg). The patient also referred asthenia, tiredness, and night fever, making it hard for her to perform daily chores 3 months before the admission.

At physical examination the patient was pale (2+/4+) and showed no signs of jaundice. Abdominal ultrasound showed a heterogeneous mass of 6.4 cm × 5.8 cm, close to the head of the pancreas; as well as thickening of the intra and extra-hepatic biliary tract, consistent with inflammation. A CT with intravenous contrast was performed, showing enlargement of periaortic lymph nodes near the hepatic hilum, with some as large as 2 cm. An abdominal MRI showed enlarged lymph nodes at the hepatic hilum, the hepatic gastric ligament, portocaval space, peripancreatic and interaortocaval regions, measuring up to 2.8 cm, leading to a main hypothesis of lymphoproliferative disease.

At EUS, the same enlarged lymph nodes were shown and EUS-guided fine needle aspiration (EUS-FNA biopsy) of the hepatic hilum was chosen for sampling with a 22-gauge needle. Histological analysis of the obtained samples showed necrotic tissue accompanied by round blast-like structures of multiple sizes and favoring fungal infection. Grocott stain confirmed the presence of fungi with morphological features consistent with *Paracoccidioides brasiliensis * (Figures 1(e) and 1(f)).

## 3. Discussion

EUS allied to fine needle biopsy is an important tool in determining diagnoses of enlarged lymph nodes, as pictured in the three cases presented here. Using a minimally invasive method, it yielded sufficient tissue for morphological analysis, and subsequent ancillary studies, confirming the diagnosis of infectious diseases in cases otherwise seen as malignant, which prompted immediate therapy with very good outcomes in all cases. Although two of these cases have already been separately illustrative of paracoccidioidomicosis diagnosis by EUS-FNA [5, 6], this is the first complete global report with discussion of the clinical, radiological and pathological findings of this fungal infection diagnosed by such a minimally invasive method, highlighting that the presence of necrosis may indicate an infectious and not a neoplastic process, especially in countries where these diseases are endemic. Before the advent of EUS, diagnoses of abdominal TB or other infectious diseases were traditionally made by percutaneous US/CT-guided FNA, but complications as hematuria, pancreatitis and pneumothorax or exploratory laparotomy were regularly reported [3, 5, 6]. The lower rate of complication in the EUS-FNA methodology compared to the percutaneous method, allied to direct visualization of the left adrenal gland makes the task of performing the procedure simple, quick, safe and easy access. It is also of particular value in patients with infectious diseases, where the critical clinical status usually precludes the use of more aggressive approaches for obtaining proper diagnostic tissue, such as laparoscopies or laparotomies [1].

The three cases reported here are also of clinical interest due to their unusual clinical presentation. Although paracoccidioidomycosis and tuberculosis are endemic in our country, they are often diagnosed due to oral or pulmonary manifestations, such as persistent cough with characteristic radiographic findings. These three cases, however, had various atypical symptoms and infectious diseases were not the primary differential diagnosis. Intra-abdominal nodes enlargement in all three cases was considered to be caused by proliferative/neoplastic disorders, such as lymphomas or metastases to lymph nodes. Using a minimally invasive procedure such as EUS-FNA, we were able to rule out malignancy and achieve histological diagnosis and start therapy immediately.

In paracoccidioidomycosis, a systemic disease with an endemic profile, intra-abdominal sites can be involved due to lymphatic and blood dissemination of the fungi, such as lymph nodes and the adrenal gland, compromised in 85%–90% of the autopsied cases [8].

Tuberculosis, on the other hand, may involve the abdomen primarily from the reactivation of a dormant focus or secondarily, when infections spread via swallowed sputum, unpasteurized milk, blood, or from an infected neighboring organ. Abdominal tuberculosis commonly affects the liver, spleen, and the ileocecal region. Pancreatic and/or peripancreatic involvement of tuberculosis, as seen in our case, is very rare, even in areas where the disease is endemic [7, 9]. The cases presented here corroborate, therefore, to the idea that infectious diseases should always be considered as a differential diagnosis for tumors located in the abdomen, especially in tropical countries.

Minimally invasive procedures capable of achieving precise diagnosis with the least possible rate of complications have an important role in current modern medicine. A wide use of cytology allied to EUS, as presented here, can aid usually critically ill patients by installing early proper therapy, without the need for aggressive interventions.

## Figures and Tables

**Table 1:** Characteristics of other cases or series published in the literature on the diagnosis of infectious diseases by endoscopy or bronchoscopy ultrasound-guided fine needle aspiration.

First author (year)	Patients, *n*	Material sampled	Diagnosis
Ahlawat (2005) [7]	01	Peripancreatic LN	Tuberculosis
Cheng (2006) [2]	01	Pancreas and mediastinal LN	Tuberculosis
Itaba (2007) [4]	01	Peripancreatic LN	Tuberculosis
Steven et al. (2011) [1]	01	Bilateral adrenal gland	Histoplasmosis
Colaiacovo (2011) [5]	01	Left adrenal gland	Paracoccidioidomycosis
Puri (2012) [3]	19	Pancreas	Tuberculosis
Silva Neto (2012) [6]	01	Hepatic hilum LN	Paracoccidioidomycosis

*n*: number of patients; LN: lymph node.
